# Neonatal Diamond‐Blackfan anemia with persistent neutropenia caused by an *RPS15A* variant

**DOI:** 10.1002/ped4.70084

**Published:** 2026-08-21

**Authors:** Hong Zheng, Kaimei Wang, Jian Wang, Jianpei Fang, Dunhua Zhou, Kunyin Qiu

**Affiliations:** ^1^ Department of Hematology/Oncology, Children's Medical Center, Sun Yat‐sen Memorial Hospital Sun Yat‐sen University Guangzhou China; ^2^ Department of Hematology/Oncology Guangdong Provincial Key Laboratory of Malignant Tumor Epigenetics and Gene Regulation, Sun Yat‐sen Memorial Hospital Sun Yat‐sen University Guangzhou China

To the Editor,

Diamond‐Blackfan anemia (DBA) is a rare inherited bone marrow failure syndrome that typically presents in infancy with macrocytic anemia, reticulocytopenia, and selective erythroid hypoplasia.^1^, ^2^ Because DBA is classically considered a disorder of isolated erythroid failure, additional cytopenias or atypical features may delay recognition. DBA is caused by haploinsufficiency of ribosomal protein genes.^3^ RPS15A, which encodes a component of the 40S ribosomal subunit, has only recently been implicated in DBA, with limited clinical data available,^4^, ^5^ and synonymous variants may disrupt mRNA splicing and escape detection by standard diagnostic approaches.^6^ Here, we describe an infant with early‐onset anemia and persistent neutropenia in whom whole‐exome sequencing (WES) was non‐diagnostic, but whole‐genome sequencing (WGS) identified a *de novo* pathogenic *RPS15A* variant, highlighting an important diagnostic pitfall.

A 1‐year‐and‐7‐month‐old child was admitted for long‐standing severe anemia and neutropenia. The patient was the first child of non‐consanguineous parents with no family history of hematologic disorders. Cytopenias were noted at birth (hemoglobin 64 g/L; white blood cell count 2.1 × 10^9^/L; normal platelets). Neonatal evaluation excluded infectious, immune‐mediated, and metabolic causes; chromosome breakage testing and telomere length were normal, and WES was non‐diagnostic. Bone marrow aspiration at 2 months showed hypocellularity with marked erythroid hypoplasia. Throughout infancy, hemoglobin remained around 40 g/L and absolute neutrophil counts fell as low as 0.1 × 10^9^/L, requiring intermittent transfusions. At 9 months, hemoglobin transiently rose to approximately 110 g/L during a documented SARS‐CoV‐2 infection before declining to baseline; neutropenia did not improve. Recurrent febrile infections developed after entering daycare. On admission, hemoglobin was 43 g/L, absolute neutrophil count 0.3 × 10^9^/L, platelet count 321 × 10^9^/L, with a reticulocyte count of 0.4 × 10^9^/L. Additional findings included macrocytosis (mean corpuscular volume 102 fL), elevated erythrocyte adenosine deaminase (1.2 IU/g Hb), and elevated fetal hemoglobin (5.0%), consistent with DBA. Physical examination showed growth retardation without craniofacial dysmorphism, thumb abnormalities, or other congenital anomalies. Bone marrow examination showed severe erythroid hypoplasia (myeloid‐to‐erythroid ratio approximately 40:1) without dysplasia or malignancy (Figure 1). WGS identified a heterozygous synonymous *RPS15A* variant (NM_001019.5:c.213G>A, p.Lys71 =), confirmed as *de novo* by Sanger sequencing. This variant was classified as pathogenic according to the ACMG/AMP 2015 criteria (PVS1+PS2+PM2+PP4+PP5). The variant affects the terminal nucleotide of exon 3, is predicted to disrupt RNA splicing, and was previously shown to cause exon skipping.^6^ Western blot analysis demonstrated markedly reduced RPS15A protein in the patient but normal expression in both parents (Figure 2), supporting haploinsufficiency. Treatment with prednisone (2 mg/kg/day) produced only partial improvement (hemoglobin 73 g/L at 6 weeks), and the addition of cyclosporine did not improve neutropenia; the patient remained transfusion‐dependent with recurrent infections (Figure S1). At the last follow‐up (age 2 years 2 months), growth remained below the 3rd percentile for age, and allogeneic hematopoietic stem cell transplantation from a matched sibling donor was planned.

**FIGURE 1:**
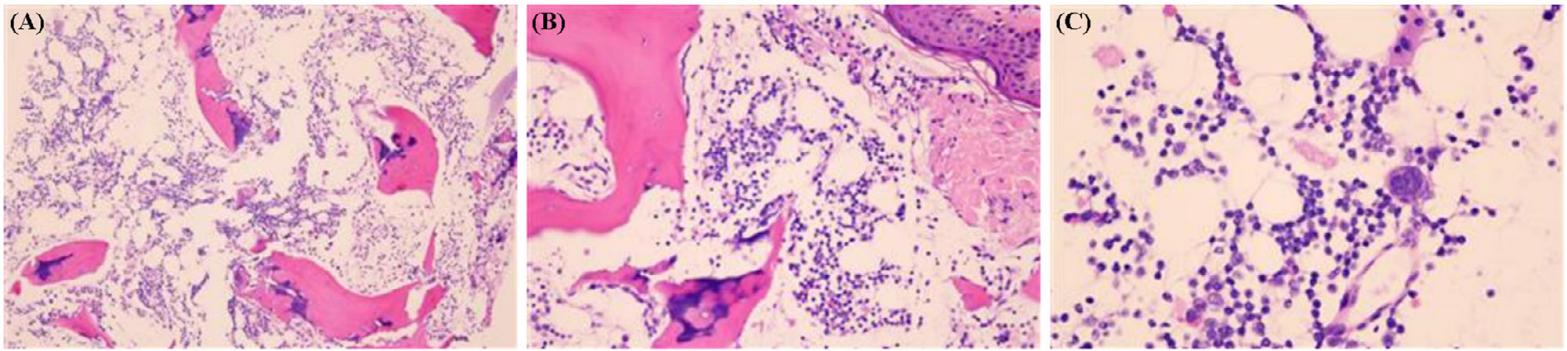
Representative bone marrow morphology in a patient with Diamond‐Blackfan anemia. (A) Hematopoietic cells account for approximately 35% of the cellularity, with an increased myeloid‐to‐erythroid ratio and no stromal fibrosis (hematoxylin counterstain, 100×); (B) Erythroid precursors are markedly decreased, predominantly at the polychromatic and orthochromatic stages (hematoxylin counterstain, 100×); (C) Lymphocytes and plasma cells are scattered throughout the marrow (hematoxylin counterstain, 200×).

**FIGURE 2:**
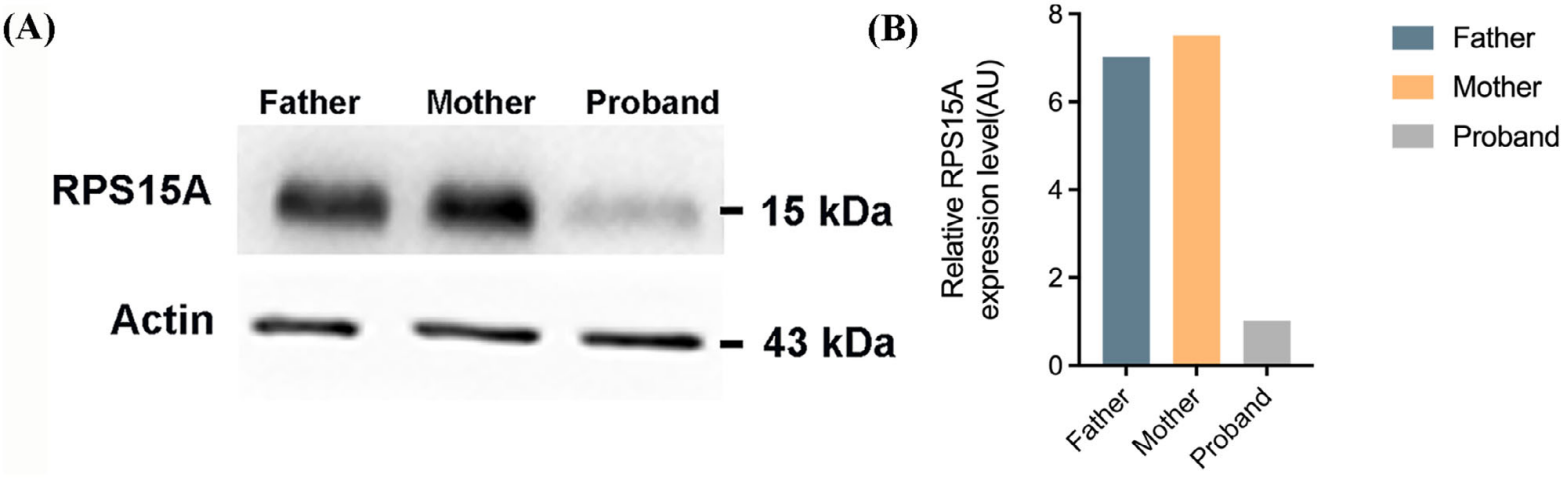
Western blot analysis of RPS15A protein expression in peripheral blood samples from the proband and the parents. (A) RPS15A protein is detectable in both parents but nearly absent in the proband. β‐actin is used as a loading control. (B) Densitometric quantification of RPS15A band intensities.

We describe an infant with DBA caused by a *de novo* *RPS15A* variant whose clinical course was marked by persistent profound neutropenia, transient hemoglobin fluctuations, and delayed genetic diagnosis. Although the patient shared the core features of DBA‐early‐onset macrocytic anemia, reticulocytopenia, preserved platelets, and erythroid hypoplasia,^2^ sustained neutropenia from birth represented a major diagnostic confounder. Our case illustrates that DBA should remain in the differential diagnosis of infants with combined cytopenias,^7^ particularly when anemia is severe, macrocytic, and present from birth. The transient hemoglobin improvement during acute viral infection may reflect increased erythropoietic drive during systemic inflammation.^8^ The absence of parallel neutrophil recovery underscores lineage‐specific vulnerability to ribosomal protein insufficiency.

Mechanistically, ribosomal protein haploinsufficiency impairs ribosome biogenesis and activates p53, inducing cell‐cycle arrest and apoptosis in erythroid progenitors.^2^ Ribosomal stress may similarly impair myeloid progenitors, and neutropenia has been reported in DBA associated with *GATA1* and *RPL35A* variants.^2^, ^9^ The near‐complete absence of RPS15A protein in our patient may have exceeded residual compensatory capacity, leading to cross‐lineage hematopoietic suppression, although this single‐case observation should be considered hypothesis‐generating. As summarized in Table S1, our patient shares the identical RPS15A c.213G>A (p.K71K) variant with the familial case reported by Ikeda et al.,^6^ yet differs in inheritance pattern (*de novo* vs. autosomal dominant), the presence of severe persistent neutropenia, suboptimal steroid response, and transfusion dependency, highlighting the phenotypic heterogeneity of RPS15A‐related DBA and underscoring that the diagnosis should not be excluded in patients without family history or congenital anomalies. A limitation is that transcript‐level analysis was not performed owing to unavailable RNA samples.

This case also illustrates the limitations of WES in inherited bone marrow failure syndromes. WGS enabled identification of the splicing variant, emphasizing the value of comprehensive genomic evaluation in unresolved cases.^10^

In summary, DBA should be considered even in the presence of persistent neutropenia or fluctuating anemia; synonymous variants may have clinically meaningful effects, and WGS is a valuable diagnostic tool when standard testing is inconclusive. Early recognition of atypical presentations of DBA may help avoid diagnostic delay and facilitate appropriate long‐term management.

## CONSENT FOR PUBLICATION

Consent for publication was obtained from the patient's parents.

## CONFLICT OF INTEREST

The authors declare no conflict of interest.

## Supporting information



Supporting Information
