# Supplementary material for: Neonatal Diamond‐Blackfan anemia with persistent neutropenia caused by an RPS15A variant
Source: Pediatr Investig. 2026 Aug 21;10(5):470–2. doi: 10.1002/ped4.70084 (PMC13495045; doi:10.1002/ped4.70084)
Supplement: Supplementary file 1 [file PED4-10-470-s001.pdf]

Supplementary Material for

Neonatal Diamond-Blackfan anemia with persistent neutropenia caused by an RPS15A variant

Hong Zheng, Kaimei Wang, Jian Wang, Jianpei Fang, Dunhua Zhou, Kunyin Qiu

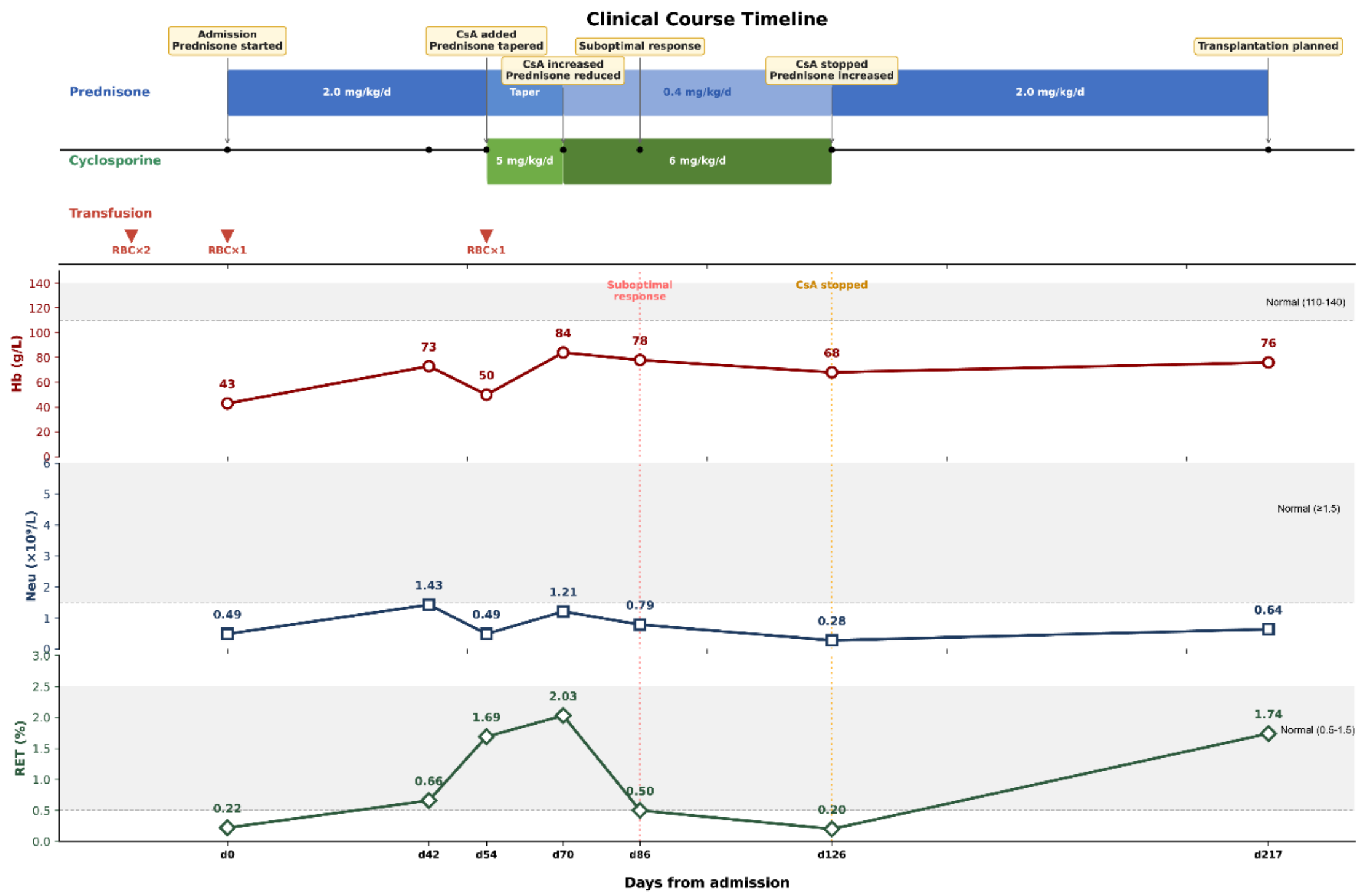

Figure S1. Clinical course timeline and therapeutic response.

Table S1. Comparison of published RPS15A-related Diamond-Blackfan anemia cases

Parameter	Case 1 (Ikeda et al., 2017)	Case 2 (Present case)
**Demographics and Clinical Features**		
Reference	Ikeda F, et al. Haematologica 2017;102:e93-e96	Present report
Sex	Female	Female
Gestational age	37 weeks	37 weeks
Birth weight	2,155 g (SGA)	2,855 g (AGA)
Birth height	47 cm	50 cm
Age at diagnosis	3 months	Delayed; genetic diagnosis after extensive workup at ~1.7 years
Current age / follow-up	14 years (at time of report)	~2 years 2 months (at last follow-up)
Congenital anomalies	Total anomalous pulmonary venous connection; bilateral acetabular dysplasia	None (no craniofacial dysmorphism, thumb abnormalities, or other physical malformations)
Growth outcomes	Not specifically reported as abnormal	Growth retardation; height and weight below 3rd percentile for age at 2 years 2 months
Family history	Positive: mother and older sister had childhood anemia; autosomal dominant inheritance	Negative: no family history of hematologic disorders; de novo mutation
**Genetic and Molecular Findings**		
Gene	RPS15A	RPS15A
Transcript	NM_001019.4	NM_001019.4
Variant (cDNA)	c.213G>A	c.213G>A
Variant (protein)	p.K71K (synonymous)	p.K71K (synonymous)
Inheritance	Familial (autosomal dominant; mother and sister affected)	De novo (confirmed absent in both parents)
Functional validation	RT-PCR confirmed aberrant splicing (exon 3 skipping); K562 cell model; zebrafish morphant model	Western blot confirmed near-complete absence of RPS15A protein; in silico splicing prediction (SpliceAI, MaxEntScan, Human Splicing Finder)
**Hematologic Features at Diagnosis**		
Hemoglobin (g/L)	88	64 (at birth); 43 (at admission, 1.7 years)
MCV (fL)	88.5 (normocytic at diagnosis; developed macrocytosis [MCV 113.0] at 14 years)	102 (macrocytosis present from admission)
WBC ($\times 10^9$/L)	5.1 (normal differential counts)	4.83 (ANC 0.3×10^9/L)

ANC ($\times 10^9$/L)	Normal (not specified)	0.3 (at admission); as low as 0.1 during infancy
Platelet count ($\times 10^9$/L)	306 (normal)	321 (normal)
Reticulocytes	0.1% (reticulocytopenia)	0.22% (reticulocytopenia)
Neutropenia	Absent (normal differential counts)	Persistent severe neutropenia from birth; ANC consistently <0.5 × 10^9/L
Erythrocyte ADA (IU/g Hb)	Not reported	1.2 (elevated)
Fetal hemoglobin (%)	Not reported	5.0% (elevated)
Bone marrow findings	Severe selective erythroid hypoplasia (0%); otherwise normal cellularity	Hypocellularity with severe erythroid hypoplasia; markedly increased myeloid-to-erythroid ratio
**Treatment and Clinical Outcomes**		
Corticosteroid therapy	Responded to initial therapy; eventually became steroid-independent	Initial response at 2 mg/kg/day (Hb improved to 73 g/L at 6 weeks); suboptimal long-term response
Cyclosporine	Not administered	Added at 5 mg/kg/day, increased to 6 mg/kg/day; discontinued at 18 weeks due to suboptimal response
Transfusion dependency	Not reported as transfusion-dependent	Yes; intermittent packed RBC transfusions required when Hb <60-70 g/L; remained transfusion-dependent at last follow-up
Infection burden	Not specifically reported	Recurrent febrile infections (respiratory and gastrointestinal); required frequent antibiotic courses; developed after entering daycare at 1 year
Viral-associated hemoglobin fluctuation	Not reported	Yes; Hb transiently increased to ~110 g/L during SARS-CoV-2 infection at 9 months, then declined to baseline
HSCT	Not mentioned	Planned; matched sibling donor identified as definitive curative strategy
Malignancy / solid tumor risk	Not mentioned at time of report (patient was 14 years old)	Under long-term surveillance; no malignancy detected at 2 years 2 months

Abbreviations: SGA, small for gestational age; MCV, mean corpuscular volume; WBC, white blood cell count; ANC, absolute neutrophil count; ADA, adenosine deaminase; HSCT, hematopoietic stem cell transplantation; RT-PCR, reverse transcriptase polymerase chain reaction.

Additional information

Whole-Exome Sequencing (WES)

WES was performed on genomic DNA extracted from peripheral blood. Genomic DNA was fragmented and subjected to library preparation, followed by target enrichment of exonic regions and flanking splice sites using the Roche KAPA HyperExome capture kit according to the manufacturer's protocol. The enriched libraries were sequenced on the MGISEQ-2000 or DNBSEQ-T7 platforms. Sequencing quality metrics included a mean target-region depth of ≥200×, with more than 98.5% of target bases covered at a depth greater than 20×. Raw sequencing reads were aligned to the UCSC hg19 human reference genome using the Burrows–Wheeler Aligner, and duplicate reads were removed prior to downstream analysis. Variant calling, including single nucleotide variants (SNVs) and small insertions/deletions (INDELs), as well as genotype determination, was performed using the Genome Analysis Toolkit following base quality score recalibration. Exon-level copy number variations were detected using ExomeDepth. Variant annotation and filtering were conducted based on the patient's clinical phenotype, population frequency databases, disease-related databases, and in silico prediction tools. Gene and variant nomenclature followed the standards of the Human Gene Nomenclature Committee and the Human Genome Variation Society, respectively. Variant pathogenicity was interpreted in accordance with the guidelines of the American College of Medical Genetics and Genomics and the Association for Molecular Pathology, with additional considerations from ClinGen and the UK Association for Clinical Genomic Science.

Whole-Genome Sequencing (WGS)

WGS was performed on genomic DNA extracted from peripheral blood. Sequencing was performed to achieve a mean coverage depth of ≥30× across the nuclear genome and ≥800× for mitochondrial DNA, with at least 98% of the genome covered at ≥10× and 90% at ≥20×. Base calling and primary data processing were conducted using a validated proprietary pipeline. Secondary bioinformatics analysis was performed using the Sentieon software suite, with sequence alignment and variant detection based on the GRCh37/hg19 reference genome. SNVs and small INDELs were annotated using the Variant Effect Predictor and interpreted with reference to multiple databases, including ClinVar, OMIM, HGMD, MITOMAP, and gnomAD, in combination with established in silico prediction algorithms; it should be noted that these databases are periodically updated and may not fully capture the most recent evidence at the time of analysis. Larger genomic alterations, including copy number variations, loss of heterozygosity, and structural variants, were identified using validated in-house algorithms based on high-throughput sequencing data, and their clinical significance was classified according to the ACMG/ClinGen technical standards. Dynamic repeat expansion mutations were analyzed using specialized algorithms to estimate trinucleotide repeat sizes and were further validated by DNA fragment analysis where applicable, with interpretation based on established literature. Variant nomenclature for SNVs and INDELs followed Human Genome Variation Society recommendations, whereas copy number and structural variants were described according to the International System for Human Cytogenomic Nomenclature. Clinical interpretation of nuclear genomic

variants and mitochondrial DNA variants was performed according to the respective ACMG guidelines.

Western Blot

Protein extracts were prepared from cells using ice-cold lysis buffer supplemented with protease inhibitors, followed by incubation on ice and clarification by centrifugation. The supernatants were collected, and protein concentrations were determined using a bicinchoninic acid (BCA) assay according to standard protocols. Equal amounts of protein were denatured in loading buffer, separated by SDS-polyacrylamide gel electrophoresis, and subsequently transferred onto polyvinylidene fluoride (PVDF) membranes. After transfer, membranes were blocked in 5% non-fat milk diluted in Tris-buffered saline containing 0.1% Tween-20 (TBST) to reduce non-specific binding. Membranes were then incubated with primary antibodies against RPS15A (proteintech.Cat No. 14942-1-AP) and β-actin (loading control) at appropriate dilutions, as specified by the manufacturers, followed by incubation with horseradish peroxidase (HRP)-conjugated secondary antibodies. After thorough washing with TBST, protein signals were visualized using an enhanced chemiluminescence (ECL) detection system and captured using an imaging platform.
